# 

**Published:** 1869-12

**Authors:** 


### CONTENTS.

ORIGINAL COMMUNICATIONS.

Annual Address before the Ohio State Dental Society .......... 493
Microscopy of a Tooth .......... 498
Amalgam, and its Proper Use .......... 502
Treatment of Tooth Pulps .......... 511
Chloro Nitrous Oxyd .......... 516
Os-Artificial Impugned .......... 519
A Peculiar Case .......... 522

PROCEEDINGS OF SOCIETIES.

American Academy of Dental Science .......... 524

SELECTIONS.

Hygiene in its Relation to Therapeutics .......... 526
Specialties .......... 530
A Key to every Imposture and Villainy .......... 532
Vegetable Nature of Diatomacæ .......... 532
Rich Beef Tea .......... 535

EDITORIAL.

Close of the Volume .......... 536
Obituary .......... 536

